# How habitat factors affect an *Aedes* mosquitoes driven outbreak at temperate latitudes: The case of the Chikungunya virus in Italy

**DOI:** 10.1371/journal.pntd.0010655

**Published:** 2023-08-17

**Authors:** Angelo Solimini, Chiara Virgillito, Mattia Manica, Piero Poletti, Giorgio Guzzetta, Giovanni Marini, Roberto Rosà, Federico Filipponi, Paola Scognamiglio, Francesco Vairo, Beniamino Caputo

**Affiliations:** 1 Departement of Public Health and Infectious Diseases, Sapienza Università di Roma, Roma, Italy; 2 Center for Health Emergencies, Fondazione Bruno Kessler, Trento, Italy; 3 Research and Innovation Centre, Fondazione Edmund Mach, San Michele all’Adige (TN), Italy; 4 Center Agriculture Food Environment, Università di Trento, San Michele all’Adige (TN), Italy; 5 Institute for Environmental Protection and Research (ISPRA), Roma, Italy; 6 Regional Service for Surveillance and Control of Infectious Diseases (SERESMI)—Lazio Region, National Institute for Infectious Diseases "Lazzaro Spallanzani" IRCCS, Rome, Italy; Faculty of Science, Ain Shams University (ASU), EGYPT

## Abstract

**Background:**

Outbreaks of *Aedes*-borne diseases in temperate areas are not frequent, and limited in number of cases. We investigate the associations between habitat factors and temperature on individuals’ risk of chikungunya (CHIKV) in a non-endemic area by spatially analyzing the data from the 2017 Italian outbreak.

**Methodology/Principal findings:**

We adopted a case-control study design to analyze the association between land-cover variables, temperature, and human population density with CHIKV cases. The observational unit was the area, at different scales, surrounding the residence of each CHIKV notified case. The statistical analysis was conducted considering the whole dataset and separately for the resort town of Anzio and the metropolitan city of Rome, which were the two main foci of the outbreak. In Rome, a higher probability for the occurrence of CHIKV cases is associated with lower temperature (OR = 0.72; 95% CI: 0.61–0.85) and with cells with higher vegetation coverage and human population density (OR = 1.03; 95% CI: 1.00–1.05). In Anzio, CHIKV case occurrence was positively associated with human population density (OR = 1.03; 95% CI: 1.00–1.06) but not with habitat factors or temperature.

**Conclusion/Significance:**

Using temperature, human population density and vegetation coverage data as drives for CHIKV transmission, our estimates could be instrumental in assessing spatial heterogeneity in the risk of experiencing arboviral diseases in non-endemic temperate areas.

## Author summary

Outbreaks of *Aedes*-borne diseases are still sporadic events in temperate Europe. Among these, a notable example is provided by the outbreak of CHIKV in Italy in 2017. We investigated which environmental variables may be spatially associated with CHIKV cases and to what extent these variables could be used to infer the occurrence of CHIKV cases when no vector data are available. To do this, we conducted a case-control study designed to assess the relative risk of observing a CHIKV case. Our results suggest that, across areas characterized by a relatively lower temperature as well as high vegetation coverage, the risk of infection is greater in the highly urbanized areas of Rome. On the other hand, in Anzio, only human population density was found to be positively associated with the likelihood of observing a CHIKV case. These preliminary findings may help identify vulnerable areas to be monitored and to be prioritized by integrated vector management as soon as the autochthonous transmission of an *Aedes*-borne disease is uncovered.

## Introduction

The impact of *Aedes*-borne viruses such as chikungunya (CHIKV) and dengue (DENV) for human public health has increased dramatically over the last 50 years, with both diseases spreading to new geographic locations [1–3]. The expansion of these diseases can be partially explained by the dispersal and proliferation of the alien mosquito species belonging to the genus *Aedes* [4]. Although large outbreaks of the *Aedes*-borne arbovirus have been rarely reported in Europe, several autochthonous transmission events have occurred in France [5,6], Croatia [7] and Italy [8,9].

Among those, two outbreaks of CHIKV transmitted by *Aedes albopictus* occurred in Italy in 2007 and 2017, representing the two largest outbreaks recorded so far in continental Europe in terms of number of cases and geographical spread [10]. The first outbreak occurred in 2007 in two rural and coastal villages (Castiglione di Ravenna and Cervia) in Northeastern Italy [11]. The second occurred in the summer of 2017, initially affecting the coastal resort town of Anzio in the Lazio Region and multiple sites within the metropolitan city of Rome and the coastal town of Latina. Secondary foci for this outbreak were identified in a coastal site of the Calabria Region (Guardavalle Marina), about 650km from Anzio [9,12].

A common practice adopted for endemic tropical areas for predicting the spatial-temporal dynamics of CHIKV and DENV cases is the use of spatio-temporal epidemiological records (i.e. number of cases, serological data) in association with eco-climatic variables (in Malaysia, [13,14]; in Philippines [15]; in Sri Lanka [16] in Thailand [17–20]; in India, [21]; in Tanzania, [22]; in Puerto Rico, [23], in Senegal [24]) rather than estimates of vector density. Several climatic factors (e.g. precipitation, temperature, relative humidity), land use (e.g. rice paddies, marshes/swamps), features of urban areas or human activities associated with potential breeding habitats, have been linked to DENV or CHIKV outbreaks.

Few examples are available for urban areas, and mostly for endemic countries, where the main arbovirus vector is *Ae*. *aegypti*. Acharya et al. [25] in Nepal for instance found a positive statistical relationship between dengue incidence and proportion of urban area and negative for proximity to roads; population density varied significantly from district to district, while the associations of land surface temperature and normalized difference vegetation index remained constant spatially. However, in non-endemic areas, as is the case of continental Europe, possibly due to the paucity of large outbreaks, similar modelling exercises have not been carried out [26].

This study employs satellite image-derived measurements and georeferenced addresses of notified human cases during the 2017 outbreak in Rome and Anzio to explore the extent to which habitat variables affect individuals’ risk of contracting CHIKV. In particular, we sought to assess: 1) which environmental variables are associated with CHIKV cases; 2) the spatial scale at which the identified association is stronger; and 3) how temperature and socio-environmental factors might influence the risk of CHIKV transmission across different environmental settings–urban (i.e. Rome) and resort town (i.e. Anzio).

## Materials and methods

For this study, a dataset was built by aggregating several population and habitat variables over a 50-meter regular grid, with a total of 11,118 geographical cells. Considered variables included temperature records (Land surface temperature), land cover classification, landscape metrics related to vegetation coverage, and human population density. The covered area encompasses both the municipality of Anzio and the metropolitan city of Rome, which are about 60km apart. GRASS GIS version 7.4.0 [27] and R version 3.4.1 [28] were used to build the spatial dataset and to conduct the proposed analysis.

### Temperature

The temperature variable (hereafter denoted as ΔLST) was calculated as the difference between Land Surface Temperature (LST) at each grid cell and LST at a reference grid cell in the Anzio municipality (41°27’00” N 12°37’51” E). This quantity, expressed in Kelvin degrees, was estimated from all the available cloud-free observations (scene cloud cover lower than 70%) at a resolution of 30m, as acquired during 2017 by TIRS thermal sensor onboard LANDSAT-8 satellite (https://earthexplorer.usgs.gov), using NDVI-derived emissivity [29] after removing residual cloud pixels using quality band masks. Temporal median statistics were then computed from time series of ΔLST. Temperature data were aggregated over time to maintain a high spatial resolution for this crucial information. Specifically, considering that high spatial resolution LST data are available from satellite data with decadal revisit time, ΔLST efficiently summarizes and describes fine spatial variability of temperature, in order to identify areas that are typically colder (e.g. natural areas) or warmer (e.g. built-up surfaces generating urban heat islands) than a reference grid cell.

### Socio-environmental variables

A land cover map was generated by considering 8 different classes based on digital multispectral aerial imagery acquired by optical sensor in the visible spectrum on May 27, 2008, and on June 27, 2008 at 0.5m spatial resolution, collected from the Italian National Geoportal (http://www.pcn.minambiente.it/GN). Mapped land cover classes were ‘bare soil’, ‘roads/concrete’, ‘building’, ‘residential building’, ‘broadleaf vegetation’, ‘coniferous vegetation’, ‘grasslands’, ‘water bodies’. Classification was obtained using SMAP (Sequential Maximum A Posteriori) supervised classification [30] in GRASS GIS software, which segments multispectral images using a spectral class model known as a Gaussian mixture distribution and spectral mean and covariance parameters. The SMAP segmentation algorithm improves the accuracy and resolution of urban mapping by segmenting the image into regions rather than segmenting each pixel separately. A total of 210 ground points were selected from a visual inspection of multispectral aerial imagery, and used for SMAP classifier training (70%) and validation (30%). Additionally, vector representation of single buildings and the road network reproduced in the Carta Tecnica Regionale (CTR), collected from the OpenData portal of Lazio Region (http://dati.lazio.it/weblist/cartografia/prodotti/2002_2003_CTRN_5K_SHP), were used as support layers to classify different areas into three mutually exclusive classes (‘roads/concrete’, ‘building’ and ‘residential building’), and to retrieve a measure of the building volume, multiplying the building plane area by the building height as provided by CTR for each building. Mapped land cover achieved an overall accuracy of 87.2% and Cohen’s kappa of 0.77. For each grid cell we computed the percentage of the three different mapped vegetation classes (ie: ‘broadleaf vegetation’, ‘coniferous vegetation’, ‘grasslands’) and the overall vegetation coverage (hereafter Veg cover) as the sum of three class percentages [31]. We further defined a qualitative variable Vegetation type representing the predominant vegetation class (*ie* above 50%, see Table 1) in the considered area. Finally, landscape metrics were computed using the R ‘landscapemetrics’ package [32] to evaluate the number and variability of vegetation patches within each grid cell, specifically the Shannon diversity index (hereafter Veg div.sh), patch richness (hereafter Vegp.rich), representing the absolute number of single vegetation patches, and the area-weighted mean shape index (hereafter Veg awmsi) [33].

**Table 1:** Summary of temperature and socio-environmental characteristics of case and control cells considering entire database.

Characteristics	Control Cell, N = 1,272	Case Cell, N = 318	p-value^*3*^
human population density (inhabitants/hectare)	10^1^ (5, 27) ^2^	14^1^ (6, 48)^2^	<0.001^3^
Vegetation coverage	40^1^ (28, 57) ^2^	34^1^ (23, 47) ^2^	<0.001^3^
Vegetation area weighted mean shape index	0.60^1^ (0.52, 0.82) ^2^	0.62^1^ (0.52, 0.81) ^2^	0.5^3^
ΔLST (°K)	4.55^1^ (2.61, 6.27) ^2^	4.34^1^ (2.67, 5.99) ^2^	0.3^3^
Vegetation Shannon diversity index	0.68^1^ (0.57, 0.85) ^2^	0.68^1^ (0.57, 0.82) ^2^	0.5^3^
Vegetation patch richness	20^1^ (13, 31) ^2^	20^1^ (13, 30) ^2^	0.7^5^
Vegetation classes			0.6^4^
0 (None)	1^6^ (<0.1%)	0^6^ (0%)	
1 (broadleaf vegetation)	36^6^ (2.8%)	11^6^ (3.5%)	
2 (coniferous vegetation)	521^6^ (41%)	139^6^ (44%)	
3 (grasslands)	714^6^ (56%)	168^6^ (53%)	
Location			>0.9^5^
Anzio	668^7^ (53%)	167^7^ (53%)	
Rome	604^7^ (47%)	151^7^ (47%)	

1 = Median, 2 = interquartile range, 3 = Wilcoxon rank sum test; 4 = Fisher’s exact test; 5 = Pearson’s Chi-squared test, 6 = Numbers of cells with Vegetation classes 0,1,2,3 (%), 7 = Number of cells in Anzio and Rome city (%).

Population data were extrapolated from population 2011 census data (http://www.istat.it) for the census sections included in the study area. Recorded population was distributed within the residential building volume of each section, in order to spatially downscale population density (inhabitants/hectare) at grid cell level. Moreover, building volume was used to calculate the adjusted population density, in order to take into account tourist and not only resident data. Average population density in residential areas, calculated using the volumetric method, was used to scale up population density in tourist-dense areas of the Anzio municipality.

### Chikungunya cases

Chikungunya case notifications within the municipalities of Rome and Anzio were collected by the Regional Service for Surveillance and Control of Infectious Diseases–Lazio Region [12]. Epidemiological data included for each identified case the geographic location of residence and the date of symptom onset range: June 27 to October 12, 2017). Details on the collection of epidemiological data are provided in [12].

### Statistical analysis

We adopted a design derived from case-control epidemiological studies. Case cells were defined as the cells of the grid where at least one CHIKV case was notified during the study period. For each “case” cell, 4 “control” cells (i.e. cells where no cases of CHIKV were notified) were randomly selected. Control cells were sampled by adopting a risk set scheme defined as follows. For any case cell, at the date of symptom onset, control cells were randomly sampled among cells without previous CHIKV cases lying within a spatial buffer of 500m from at least one case cell. The distance of 500m was chosen according to evidence provided by a modeling study, which highlighted that 90% of CHIKV transmission chains likely occurred within this distance from the index case [34]. Control cells were randomly sampled from those associated with residential locations (i.e. by discarding cells that according to data provided by the Italian National Statistics Institute contained a zero value human density population). The final dataset was composed of 318 case cells (167 in the Anzio municipality and 151 in the Rome municipality) and 1,272 control cells. In each cell, we first calculated the mean value of environmental variable buffers from 0 to 250m. The maximum buffer radius was chosen to avoid a potential overlap between the case and control cells buffers. Moreover, this distance is consistent with the expected flight range (estimated by capture-mark-recapture data) of 95% of *Ae*. *albopictus* adult females in 3 days [35]. Descriptive statistics were carried out with Wilcoxon rank, Fisher’s and Pearson’s Chi-squared tests as appropriate.

We used a conditional logistic regression model to explore the association between ΔLST, population density and environmental variables at different spatial scales and CHIKV notified cases. We included a priori in the multivariate model the ΔLST and population density variables at the spatial scale of the CHIKV cases. However, to identify the most suitable spatial scale for the considered environmental variables, we investigated the univariate relationships between each of the five environmental variables (Shannon diversity index, patch richness, area-weighted mean shape index, vegetation coverage, and vegetation type) at six geographical levels (50m increasing circular buffers as explained above) and the logit of detecting a case, and selected the final spatial scale associated with the best model performance, based on the Akaike Information Criterion (AIC). Then we included in the multivariate model only the environmental variables having a p-value smaller than 0.2 [36]. We also entered a categorical term for case location (Anzio or Rome). Vegetation coverage was also entered after categorization in 4 quartiles to facilitate the interpretation of interaction terms with population density. Finally, interaction between ΔLST and location (Anzio or Rome) was considered to capture potential differences in the LSTs between the two areas. We also fitted separate regression models for each location.

Alternative regression models were considered for sensitivity analysis. First, we added cubic spline terms, with 3 knots, for longitude and latitude of cell centroids instead of using a categorical variable for the location (Anzio or Rome). Second, we adjusted the population size of Anzio to account for people residing in Anzio during summer months. Finally, for the environmental variable having p-value<0.2 we used a quantitative variable rather than categorizing it in 4 quartiles.

## Results

**Fig 1:**
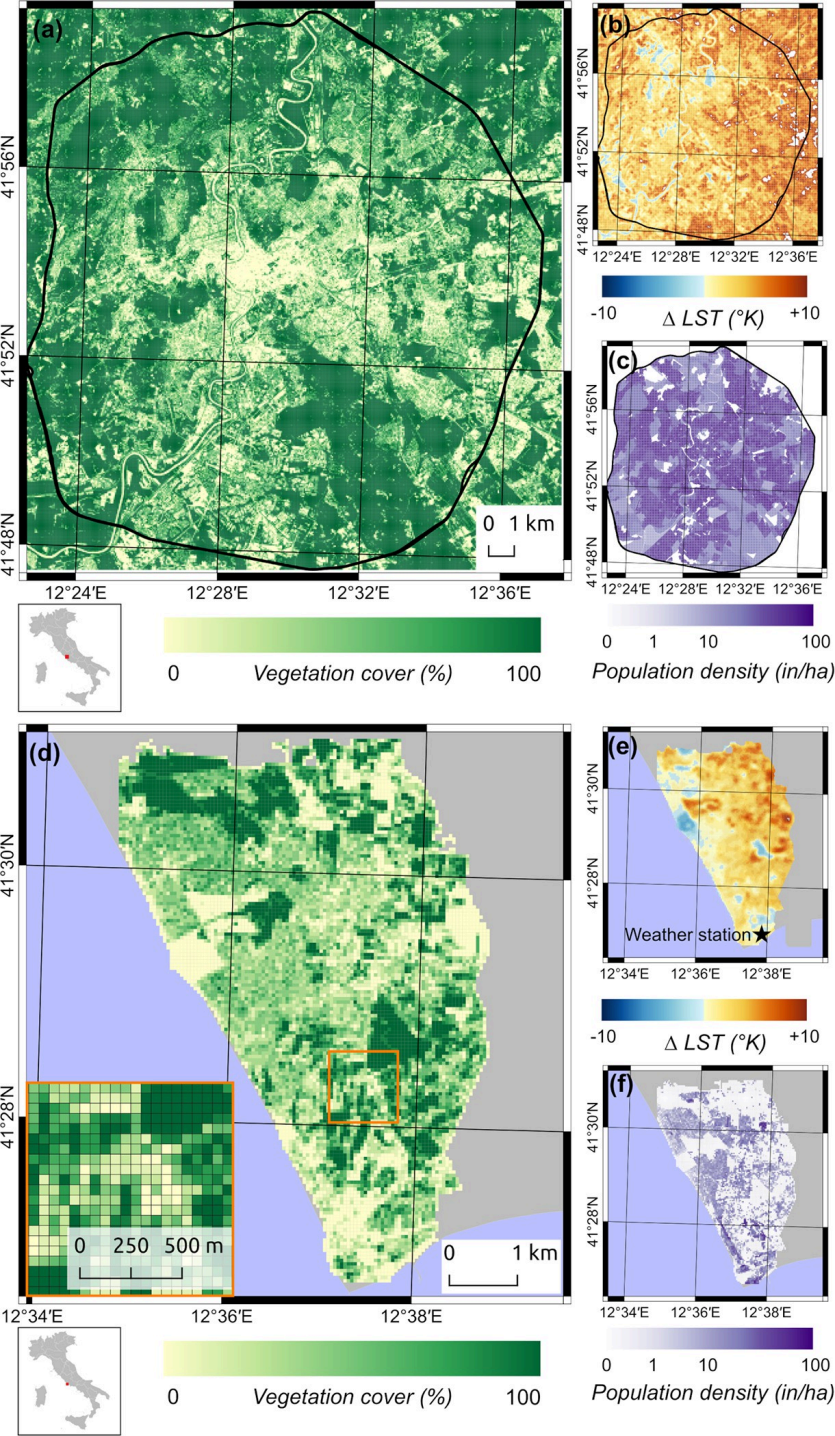
**Color scales at grid cell level (50m) of vegetation coverage in percentage terms in Rome (a) and Anzio (d); ΔLST in Kelvin degrees (°K) in Rome (b) and Anzio (e); population density in inhabitants/hectare (in/ha) in Rome (c) and Anzio (f)**. Shapefile republished from GADM database (https://gadm.org/download_country_v3.html) under a CC BY license of Global Administrative Areas (GADM), copyright 2018–2022.

Temperature and socio-environmental characteristics of case and control cells in Anzio and Rome are shown in Table 1 and Fig 1. Compared to control cells, case cells show a higher median population density and lower vegetation coverage (Fisher’s exact test, p-value<0.001 Table 1). A similar number of CHIKV cases was reported from Anzio (N = 167) and Rome (N = 151), although population density was higher in Rome (min = 1.92 max = 503, inhabitants/hectare) compared to Anzio (min = 1.36 max = 92 inhabitants/hectare), and the minimum and maximum median ΔLST were -0.69 and 12.47°K and -1.34 and 8.78°K in Rome and Anzio respectively (Fig 1).

The results of univariate logistic models linking environmental variables and the risk of detecting a case are shown in Tables 2 and S1. Table 2 shows the univariate model with the lowest AIC, among the 6 buffers tested for each covariate (from 0 to 250m, range 50m). For most considered spatial scales (from 0m to 250m buffer), environmental variables (ie Vegetation Shannon diversity index, Vegetation area weighted mean shape index, Vegetation patch richness, Vegetation classes) were not significantly associated with a CHIKV occurrence (Table 2, p-values<0.2). The only exception was found for vegetation coverage at the smallest spatial scale (0-50m from the centroid of the cell covering the residence of a case) (Table 2, p-value<0.001).

**Table 2:** Summary of univariate regression models. Spatial scale of 5 environmental variables associated with the logit of detecting notified CHIKV cases in the entire dataset (Anzio&Rome = 318 CHIKV cases).

Variables	scale	coef	Se coef	p.value	AIC
Vegetation coverage	0-50mt	-0.022	0.003	<0.0001	1154.83
Vegetation Shannon diversity index	200-250mt	-0.194	0.434	0.654	1199.39
Vegetation area weighted mean shape index	200-250mt	0.11	0.358	0.758	1162.05
Vegetation patch richness	0-50mt	-0.003	0.005	0.638	1199.37
Vegetation classes	0-50mt	-0.115	0.109	0.292	1198.48

Coef = Coefficient estimates by regression model. SE coef = Standard Error.

Based on the results of habitat variables, we entered Vegetation coverage as covariate in the multivariate model (Table 3).

**Table 3:** Relationship between temperature/socio-environmental variables and notified CHIKV cases resulting from the entire dataset.

Variables	OR (CI 95%)
human population density	1.007 (1.0007–1.0137)
Vegetation coverage (II quart)	0.912 (0.578–1.439
Vegetation coverage (III quart)	0.812 (0.507–1.298)
Vegetation coverage (IV quart)	0.185 (0.098–0.348)
ΔLST	0.954 (0.830–1.097)
Rome	3.569 (1.4009–9.097)
human population density*Vegetation coverage (II quart)	0.996 (0.987–1.006)
human population density*Vegetation coverage (III quart)	0.996 (0.985–1.007)
human population density*Vegetation coverage (IV quart)	1.017 (1.000–1.035)
ΔLST *Rome	0.824 (0.690–0.984)

(OR: odds ratio, lower and upper limits of 95% confidence interval). II quart = Second quartile, III quart = third quartile, IV = fourth quartile.

We found a slightly higher risk of CHIKV infection in populated and vegetated cells, as cells that fall in the 4^th^ quartile of vegetation coverage had a 1.8% (CI 95%: 0.01%. 3.5%) increase in the risk of notification of a CHIKV case during the study period. CHIKV risk is positively associated with ΔLST (i.e. cells that are cooler compared to reference cells have a higher probability of harboring CHIKV transmission) only in Rome (Table 3).

In sensitivity analyses we found similar results when using natural splines of geographical coordinates of cell centroids to adjust for location instead of using a categorical variable for location (S2 Table) or when adjusting the population density of Anzio to account for increased density during summer months (S3 Table). Finally, we found similar results when using quantitative vegetation coverage variable (S4 Table).

**Fig 2:**
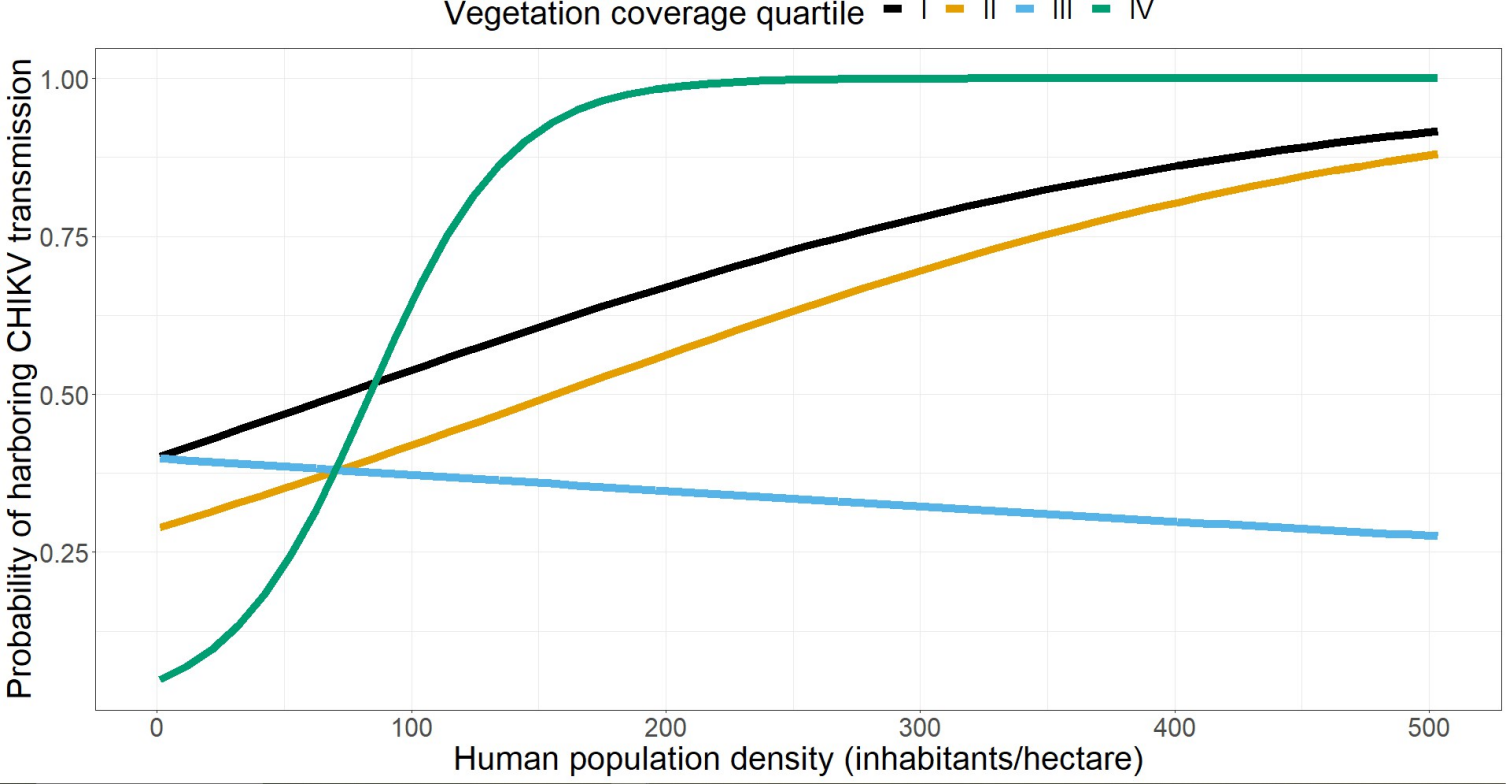
Estimated probability of a cell harboring CHIKV transmission in Rome using a conditional regression model.

The same multivariate model applied to the entire dataset was refitted for Anzio and Rome separately (Table 4). In Rome we found significant associations between the risk of CHIKV transmission with higher population density, vegetation coverage (Fig 2) and cooler cells. The model for Anzio shows a significant interaction term between human density population and vegetation coverage (p-value<0.001). However, a variance analysis (anova with LRT test, p-value = 0.16) conducted to compare model performances when the interaction term is included (or not) in the regression showed no statistical support to include the interaction term (we obtained the opposite result when comparing the performance of different models applied to Rome data only).

**Table 4:** Relationship between temperature/socio-environmental variables and notified CHIKV cases resulting from Rome (N cases = 151) and Anzio (N cases = 167) data separately.

Location	Variables	OR (CI 95%)
Rome	human population density	1.00559 (0.99837–1.0129)
	vegetation coverage (II quartile)	0.60689 (0.23835–1.5453)
	vegetation coverage (III quartile)	0.99406 (0.41592–2.3759
	vegetation coverage (IV quartile)	0.07242 (0.02504–0.2095)
	ΔLST	0.72214 (0.61043–0.8543)
	human population density * vegetation coverage (II quartile)	1.00020 (0.98787–1.0127)
	human population density * vegetation coverage (III quartile)	0.99334 (0.97939–1.0075)
	human population density * vegetation coverage (IV quartile)	1.03115 (1.00857–1.0542)
Anzio	human population density	1.033 (1.0035–1.0634)
	vegetation coverage (II quartile)	0.8158 (0.3986–1.6699)
	vegetation coverage (III quartile)	1.3595 (0.5801–3.1861)
	vegetation coverage (IV quartile)	0.4264 (0.1102–1.6502)
	ΔLST	0.9204 (0.7508–1.1283)
	human population density * vegetation coverage (II quartile)	1.0085 (0.9559–1.0639)
	human population density * vegetation coverage (III quartile)	0.9015 (0.8141–0.9982)
	human population density * vegetation coverage (IV quartile)	0.9444 (0.7589–1.1752)

(OR: odds ratio, lower and upper limits of 95% confidence interval). CI = confidence intervals.

## Discussion

Our results suggest that land cover and population density represent key predictors not only for vector abundance [37–40] but also for CHIKV transmission risks. This result was shown by the model considering data from both areas (Anzio and Rome) where locations with similar vegetation coverage (i.e. in same quartile) but higher population density had a higher risk of observing notifications of CHIKV cases. Indeed, after adjusting for other factors it is more probable that a case will reside in a highly populated area. However, we showed that the increase in risk associated with a higher population density was steeper in locations characterized by a larger vegetation coverage. Therefore, after adjusting for temperature differences, the occurrence of CHIKV cases is positively associated with the presence of highly vegetated small buffers within large populated areas. Interestingly, when considering Rome and Anzio separately, this association was maintained only in the metropolitan area. Probably, this result is due to the non-homogenous landscape characterizing the urban texture of the city of Rome, that induces mosquitoes to aggregate within and around suitable green areas such as parks and villas. Our hypothesis is that vegetation-covered areas in cities are probably watered regularly creating anthropogenic breeding sites for the oviposition and development of aquatic stages [21,41] while also being suitable habitats for biting and resting adult mosquitoes. Moreover, the high human density surrounding places used for outdoor activities during the daily biting activity of *Ae*. *albopictus* may increase human-vector encounters. This hypothesis is supported by previous evidences reported by Samson et al., 2013 [42] showing that *Ae*. *albopictus* rests during the daytime in the vegetation around residential areas, thus demonstrating that landscape can influence spatial distribution and behavior. Moreover, previous studies showed the importance of vegetation in urban areas also for what concerns to outdoor resting preferences [40,43], high plasticity in feeding behaviour [44] and rapid active dispersal [45] of *Ae*. *albopictus*. In contrast with Rome, Anzio is characterized by a homogenous presence of townhouses and villas with small gardens, which represent an ideal habitat for *Aedes* mosquito species. Similarly, temperature-related effects were observed in Rome but not in Anzio (i.e. higher odds of observing CHIKV cases in cooler cells). It is likely that in heavily urbanized areas such as the metropolitan city of Rome, on average warmer cells are less favorable for the vector, probably due to the morphology of the landscape not captured by vegetation coverage alone.

Our work analysed which factors may have had an impact on the occurrence of CHIKV cases during the 2017 outbreak. Our findings on the association between highly anthropized areas, vegetated area and the likelihood of observing cases are consistent with those coming from endemic countries such as Tanzania [22], Senegal [24] and Sri Lanka [16], but not from Brazil where socioeconomic conditions played a greater role than green areas in characterizing a chikungunya outbreak [46]. However, it is important to note that in non-endemic countries (*i*.*e*. continental Europe) the probability of observing an exotic arbovirus outbreak as well as its final size are driven also by other variables such as the timing and frequency of arboviral introduction from endemic countries through infected travellers. The two areas first impacted by the 2017 outbreak (Rome and Anzio) presented the optimal conditions for experiencing an arbovirus outbreak. Rome, hosts the major airline hub in Italy, the international airport of Rome Fiumicino where the probability of introduction of infected travellers is not neglectable [47]. On the other hand, Anzio is a seaside location devoted to domestic tourism and characterized by favourable urban habitats for *Ae*. *albopictus*. Many people commute from here to Rome (~60km) or other cities in the region on a daily basis therefore enhancing the probability of further seeding local transmission [12]

The following limitations should be taken into consideration to interpret our results. Firstly, remote sensing imagery can provide only a partial overview of the ecological characteristic of a site with respect to its potential suitability for a mosquito population as it is currently unable to assess the presence and conditions of potential breeding sites. Unfortunately, gathering information on breeding sites or the type of habitat throughout just a small part of the outbreak area (Rome = 1.200 km^2^, Anzio = 43 km^2^) would have required extensive and costly field survey, currently unfeasible for public health authorities. Moreover, the studied environmental variable, vegetation coverage, despite being frequently used [37–40], is an aggregate measure and therefore may provide only partial information on the type of habitat that is more suitable for *Ae*. *albopictus*. Indeed, the high plasticity and adaptability to human and natural environment of this species pose a challenge in reducing the assessment of its habitat suitability to just few indicator variables. Secondly, we do not have information on socio-economic conditions and differences in our study area. It is important to note that socio-economic and demographic indices (*ie*. Stegomyia indices) have previously been related to dengue and chikungunya cases [46,48,49] despite competing evidence suggesting a lack of reliability [50]. We may assume that in Anzio, socio-economic conditions were homogeneous, but they may have been an important factor in Rome. Thirdly, we assumed that the rate of case reporting was homogeneous over time and different residential area. If this assumption was not met, then our estimates would be biased toward area with higher reporting rate. Finally, we assumed that the infection was contracted at the geographic coordinates of residence, while the individual may have acquired the infection in other locations (e.g., at work, at school or while commuting). The rationale behind this simplifying assumption is based on previous modelling results [34], suggesting that transmission mostly occurs within 500 meters of the residence of primary infectors.

Despite these study limitations, these results suggest that within metropolitan areas, the coexistence of specific environmental characteristics may increase the risk of CHIKV transmission. Our study represents one of the first attempts to explore the relationship between habitat variables and the occurrence of CHIKV cases in non-endemic areas and may represent a step forward towards the assessment of possible factors influencing the transmission of CHIKV at a micro-geographical scale. In addition, the use of satellite-derived measurements in the considered approach offers the possibility of scaling up the developed approach to other parts of the country and help prioritize surveillance effort and epidemiological investigation whenever arboviral circulation is suspected.

## Supporting information

S1 Table Spatial scale of 5 environmental variables associated to logit of detecting CHIKV notified cases in the entire dataset. Table shows the univariate model among the 6 buffers tested for each covariate (from 0 to 250mt, range 50mt).
(DOCX)

S2 Table Relationship between temperature/socio-environmental variables and notified CHIKV cases resulting from the entire dataset using natural spline for longitude and latitude of cell centroids (OR: odds ratio, lower and upper limits of 95% confidence interval). (DOCX)

S3 Table Relationship between temperature/socio-environmental variables and notified CHIKV cases resulting from the entire dataset using natural spline for longitude and latitude of cell centroids (OR: odds ratio, lower and upper limits of 95% confidence interval) and adjusted population for Anzio. (DOCX)

S4 Table Relationship between temperature/socio-environmental variables and notified CHIKV cases resulting from the entire dataset using quantitative vegetation coverage variable (OR: odds ratio, lower and upper limits of 95% confidence interval). (DOCX)
