# Supplementary material for: How habitat factors affect an Aedes mosquitoes driven outbreak at temperate latitudes: The case of the Chikungunya virus in Italy
Source: PLoS Negl Trop Dis. 2023 Aug 17;17(8):e0010655. doi: 10.1371/journal.pntd.0010655 (PMC10465128; doi:10.1371/journal.pntd.0010655)
Supplement: S1 Table — Table shows the univariate model among the 6 buffers tested for each covariate (from 0 to 250mt, range 50mt). (DOCX) [file pntd.0010655.s001.docx]

**S1 Table**: Spatial scale of 5 environmental variables associated to logit of detecting CHIKV notified cases in the entire dataset, Table shows the univariate model among the 6 buffers tested for each covariate (from 0 to 250mt, range 50mt).

| Variables | scale | Coef | Se Coef | p,value | AIC |
| --- | --- | --- | --- | --- | --- |
| Vegetation coverage | 0m | -0,022 | 0,003 | <0,0001 | 1154,83 |
|  | 50m | -0,018 | 0,004 | <0,0001 | 1179,86 |
|  | 100m | -0,016 | 0,005 | 0,001 | 1187,42 |
|  | 150m | -0,016 | 0,005 | 0,002 | 1189,24 |
|  | 200m | -0,016 | 0,006 | 0,003 | 1190,39 |
|  | 250m | -0,014 | 0,006 | 0,012 | 1193,11 |
| Vegetation Shannon diversity index | 0m | -0,11 | 0,263 | 0,675 | 1199,41 |
|  | 50m | -0,146 | 0,346 | 0,672 | 1199,41 |
|  | 100m | -0,058 | 0,386 | 0,88 | 1199,56 |
|  | 150m | -0,097 | 0,411 | 0,813 | 1199,53 |
|  | 200m | -0,171 | 0,424 | 0,688 | 1199,43 |
|  | 250m | -0,194 | 0,434 | 0,654 | 1199,39 |
| Vegetation area weighted mean shape index | 0m | 0,014 | 0,266 | 0,957 | 1199,14 |
|  | 50m | -0,01 | 0,32 | 0,975 | 1193,69 |
|  | 100m | -0,002 | 0,336 | 0,996 | 1193,24 |
|  | 150m | 0,107 | 0,348 | 0,758 | 1173,99 |
|  | 200m | 0,073 | 0,356 | 0,837 | 1167,68 |
|  | 250m | 0,11 | 0,358 | 0,758 | 1162,05 |
| Vegetation patch richness | 0m | -0,003 | 0,005 | 0,638 | 1199,37 |
|  | 50m | 0,003 | 0,006 | 0,658 | 1199,39 |
|  | 100m | 0,002 | 0,007 | 0,749 | 1199,48 |
|  | 150m | 0,002 | 0,007 | 0,741 | 1199,48 |
|  | 200m | 0,002 | 0,007 | 0,775 | 1199,51 |
|  | 250m | 0,002 | 0,007 | 0,783 | 1199,51 |
| Vegetation classes | 0m | -0,115 | 0,109 | 0,292 | 1198,48 |
|  | 50m | -0,082 | 0,118 | 0,486 | 1199,1 |
|  | 100m | -0,069 | 0,122 | 0,57 | 1199,27 |
|  | 150m | -0,064 | 0,125 | 0,609 | 1199,33 |
|  | 200m | -0,053 | 0,126 | 0,67 | 1199,41 |
|  | 250m | -0,051 | 0,126 | 0,683 | 1199,42 |
