# Supplementary material for: How habitat factors affect an Aedes mosquitoes driven outbreak at temperate latitudes: The case of the Chikungunya virus in Italy
Source: PLoS Negl Trop Dis. 2023 Aug 17;17(8):e0010655. doi: 10.1371/journal.pntd.0010655 (PMC10465128; doi:10.1371/journal.pntd.0010655)
Supplement: S2 Table — (DOCX) [file pntd.0010655.s002.docx]

**S2 Table**: Relationship between temperature/socio-environmental variables and notified CHIKV cases resulting from the entire dataset using natural spline for longitude and latitude of cell centroids (OR: odds ratio, lower and upper limits of 95% confidence interval).

| **Variables** | **OR (CI 95%)** |
| --- | --- |
| population density | 1,007 (1-1,013) |
| Vegetation coverage (II quart) | 0,853 (0,540-1,348) |
| Vegetation coverage (III quart) | 0,736 (0,462-1,174) |
| Vegetation coverage (IV quart) | 0,174 (0,092-0,331) |
| ΔLST | 0,821(0,732-0,921) |
| population density*Vegetation coverage (II quart) | 0,997 (0,988-1,007) |
| population density*Vegetation coverage (III quart) | 0,997 (0,987-1,008) |
| population density*Vegetation coverage (IVI quart) | 1,017 (0,995-1,036) |
