# Supplementary material for: How habitat factors affect an Aedes mosquitoes driven outbreak at temperate latitudes: The case of the Chikungunya virus in Italy
Source: PLoS Negl Trop Dis. 2023 Aug 17;17(8):e0010655. doi: 10.1371/journal.pntd.0010655 (PMC10465128; doi:10.1371/journal.pntd.0010655)
Supplement: S3 Table — (DOCX) [file pntd.0010655.s003.docx]

**S3 Table**: Relationship between temperature/socio-environmental variables and notified CHIKV cases resulting from the entire dataset using natural spline for longitude and latitude of cell centroids (OR: odds ratio, lower and upper limits of 95% confidence interval) and adjusted population for Anzio.

| **Variables** | **OR (CI 95%)** |
| --- | --- |
| Total adj population density | 1,007 (1,000-1,014) |
| Vegetation coverage (II quart) | 0,951 (0,596-1,519) |
| Vegetation coverage (III quart) | 0,829 (0,511-1,344) |
| Vegetation coverage (IV quart) | 0,1852(0,097-0,351) |
| ΔLST | 0,964(0,840-1,108) |
| Roma | 3,772 (1,499-9,493) |
| Total adj population density * Vegetation coverage (II quart) | 0,995(0,986-1,005) |
| Total adj population density * vegetation coverage (III quart) | 0,996(0,985-1,006) |
| Total adj population density * vegetation coverage (IV quart) | 1,01 (1,000-1,036) |
| ΔLST*Roma | 0,815(0,683-0,972) |
