# Supplementary material for: How habitat factors affect an Aedes mosquitoes driven outbreak at temperate latitudes: The case of the Chikungunya virus in Italy
Source: PLoS Negl Trop Dis. 2023 Aug 17;17(8):e0010655. doi: 10.1371/journal.pntd.0010655 (PMC10465128; doi:10.1371/journal.pntd.0010655)
Supplement: S4 Table — (DOCX) [file pntd.0010655.s004.docx]

**S4 Table**: Relationship between temperature/socio-environmental variables and notified CHIKV cases resulting from the entire dataset using quantitative vegetation coverage variable (OR: odds ratio, lower and upper limits of 95% confidence interval).

| **Variables** | **OR (CI 95%)** |
| --- | --- |
| population density | 1.0011 (0.9947-1.0076) |
| Vegetation coverage | 0.9721 (0.9636-0.9807) |
| ΔLST | 0.9100 (0.7934-1.0438) |
| Roma | 2.6986 (1.1022-6.6070) |
| population density * vegetation coverage | 1.0002 (1.000-1.0004) |
| ΔLST*Roma | 0.8650 (0.7276-1.0282) |
